# Comparative Efficacy and Toxicity of Weekly Carboplatin Versus Cisplatin in Concurrent Chemoradiotherapy for Locally Advanced Head and Neck Squamous Cell Carcinoma: A Quasi-experimental Study From Bangladesh

**DOI:** 10.7759/cureus.96586

**Published:** 2025-11-11

**Authors:** Ahammad Al Mamun Sweet, Md. Ershadul Haque, Md. Abdul Mannan, Md. Ruhul Amin Bhuiyan, Aditi Paul Chowdhury, Khandaker Md. Rezwan Bayzid, A. Z. M. Sumsuzoha, Muhammad Adnan Arifeen

**Affiliations:** 1 Department of Clinical and Radiation Oncology, Labaid Cancer Hospital and Super Speciality Centre, Dhaka, BGD; 2 Department of Radiotherapy, Rangpur Medical College Hospital, Rangpur, BGD; 3 Department of Oncology, North East Medical College and Hospital, Sylhet, BGD; 4 Department of Oncology, Ahsania Mission Cancer and General Hospital, Dhaka, BGD; 5 Department of Oncology, Thengamara Mohila Sabuj Sangha (TMSS) Medical College and Rural Clinical Hospital (RCH), Bogura, BGD

**Keywords:** carboplatin, cisplatin, concurrent chemoradiotherapy, head and neck squamous cell carcinoma, toxicity profile

## Abstract

Background

Cisplatin-based concurrent chemoradiotherapy (CCRT) is the standard care for locally advanced squamous cell carcinoma of the head and neck (LA-SCCHN). However, its toxicity limits use, especially in resource-limited settings. Carboplatin, with less toxicity, is a potential alternative, yet comparative South Asian evidence is scarce. This study compared the efficacy and toxicity of weekly carboplatin versus cisplatin, concurrently with radiotherapy in patients with LA-SCCHN.

Methodology

This quasi-experimental study was conducted at a tertiary care hospital in Bangladesh. A total of 90 patients with histologically confirmed LA-SCCHN were non-randomly allocated to receive either weekly cisplatin (40 mg/m²) or weekly carboplatin (area under the curve (AUC)-2), concurrently with external beam radiotherapy (66 Gray over 6.5 weeks). Treatment response was evaluated using Response Evaluation Criteria in Solid Tumors version 1.1, and toxicity was graded per Common Terminology Criteria for Adverse Events version 3.0. Statistical analysis was performed using SPSS version 25 (IBM Corp., Armonk, NY, USA), with a p-value <0.05 considered significant.

Results

Both cisplatin and carboplatin demonstrated comparable complete response rates of 17 (37.8%) versus 18 (40.0%) at six weeks, 18 (40.0%) versus 20 (44.4%) at 12 weeks, and 19 (42.2%) versus 22 (48.9%) at 24 weeks, with statistically insignificant differences. Cisplatin was associated with higher rates of mucocutaneous and gastrointestinal toxicities, while carboplatin was linked to increased hematologic toxicity, particularly thrombocytopenia (p = 0.040). Late toxicities were similar across groups.

Conclusions

Weekly carboplatin demonstrated comparable efficacy to weekly cisplatin, with radiotherapy for LA-SCCHN, with a distinct toxicity profile. Carboplatin may be a clinically viable alternative for patients unsuitable for cisplatin, particularly in settings with limited supportive care.

## Introduction

Head and neck cancer (HNC) represents a major global health burden and ranks among the most common malignancies worldwide [1]. According to GLOBOCAN 2020, approximately 890,000 new HNC cases were reported worldwide, accounting for 4.5% of all cancer diagnoses across genders and age groups. The corresponding mortality was nearly 450,000 deaths, representing 4.6% of all cancer-related fatalities [2]. These figures highlight the significant morbidity and mortality associated with HNC, especially in low- and middle-income countries where late-stage presentation and limited access to specialized care worsen outcomes [1].

Squamous cell carcinoma of the head and neck (SCCHN) is the predominant subtype, typically arising from the mucosal epithelium of the oral cavity, oropharynx, hypopharynx, or larynx [3]. SCCHN is usually locoregional at presentation, requiring a multidisciplinary approach, including surgery, radiotherapy, and chemotherapy [4]. For patients with locally advanced, non-metastatic disease (stages III-IVB), concurrent chemoradiotherapy (CCRT) is the standard of care [4]. The addition of chemotherapy to radiotherapy enhances radiosensitivity, improves locoregional control, and provides a modest but clinically meaningful survival benefit [5].

Cisplatin, the most widely used radiosensitizer in SCCHN CCRT regimens, has proven efficacy but is frequently limited by significant toxicity [6]. It is associated with nephrotoxicity, neurotoxicity, ototoxicity, severe nausea and vomiting, and myelosuppression [6,7]. Such toxicities often lead to dose reductions, treatment delays, or discontinuation, ultimately compromising outcomes [8]. Patients with renal impairment, advanced age, or poor performance status are frequently ineligible for cisplatin-based therapy.

Carboplatin, a second-generation platinum analog, has emerged as a potential alternative in the CCRT setting [9]. Although carboplatin shares a similar mechanism of action, DNA cross-linking and apoptosis induction, its toxicity profile is generally more favorable [10]. It is less nephrotoxic, neurotoxic, and emetogenic than cisplatin and does not require intensive hydration, making it more suitable for outpatient administration [10]. Its principal toxicity, however, is hematologic suppression, particularly thrombocytopenia [11]. Despite these advantages, evidence supporting carboplatin substitution remains inconclusive, particularly with weekly dosing regimens.

In Bangladesh, HNC poses a major oncologic challenge. Although comprehensive national statistics are limited, institutional data provide important insights. The National Institute of Cancer Research and Hospital (NICRH) Cancer Registry (2017) reported that HNCs accounted for 10.5% of all malignancies, most commonly involving the oral cavity, hypopharynx, and oropharynx. Despite this high prevalence, access to timely diagnosis and treatment is hindered by limited infrastructure, financial barriers, and low public awareness [12]. The primary objective of this study was to compare treatment efficacy between weekly cisplatin and carboplatin, measured by tumor response at 6, 12, and 24 weeks post-treatment using the Response Evaluation Criteria in Solid Tumors version 1.1 (RECIST v1.1). The secondary objectives were to compare acute and late treatment-related toxicities between the two groups, graded according to the Common Terminology Criteria for Adverse Events version 3.0 (CTCAE v3.0).

## Materials and methods

Study design and setting

This quasi-experimental study was conducted in the Department of Oncology at Khwaja Yunus Ali Medical College and Hospital (KYAMCH) in Enayetpur, Sirajganj, Bangladesh, over 18 months from June 2019 to December 2020. The primary objective was to compare the efficacy (tumor response) and toxicity profiles of weekly carboplatin and weekly cisplatin when administered concurrently with radiotherapy in patients with locally advanced SCCHN.

Patient selection

Patients were enrolled through purposive sampling from the Outpatient Department of Oncology at KYAMCH. Those who met the below-mentioned inclusion criteria were enrolled consecutively.

Inclusion Criteria

Histologically confirmed primary SCCHN; stage III or IV disease without distant metastasis, diagnosed with clinical examination and imaging (CT/MRI and/or PET-CT); age 18-70 years; Eastern Cooperative Oncology Group (ECOG) performance status 0-2; and adequate hematologic, renal, and hepatic function. Performance status was assessed using the ECOG scale [13,14].

Exclusion Criteria

Prior surgery, radiotherapy, or chemotherapy; multiple primaries; uncontrolled comorbidities (e.g., diabetes, cardiovascular or renal disease); psychiatric illness; pregnancy or lactation; or incarceration.

Sample size calculation

Sample size was calculated based on the primary outcome of treatment response using proportions reported by Dutta et al. [15], who observed response rates of 51% for carboplatin and 22% for cisplatin. Assuming a two-sided test with a significance level (α) of 0.05 and 80% power, the calculated sample size was 82 patients. To account for a potential 10% loss to follow-up, the final target sample size was increased to 90 patients, with 45 participants allocated to each treatment arm.

$n=\frac{P_1(1-P_1 )+P_2(1-P_2 )}{(P_1-P_2 )^2}(Z_\alpha+Z_\beta)^2$

Treatment protocols

Patients were assigned non-randomly to two treatment arms. Chemotherapy began on the first day of radiotherapy and continued weekly for six cycles, with clinical fitness assessments before each cycle. Standard safety precautions were followed during drug handling, including careful transfusion with protective gloves. Both cisplatin and carboplatin were sourced from Drug International Ltd., Bangladesh, to ensure consistent drug quality.

Arm A (Cisplatin)

Weekly cisplatin (40 mg/m²) in 500 mL of normal saline over two hours. Prehydration: 500 mL of normal saline with corticosteroids, antiemetics, and H₂ blockers over one hour. Posthydration: 1 L of normal saline with 20 mg furosemide over two hours.

Arm B (Carboplatin)

Weekly carboplatin (area under the curve (AUC)-2) in 500 mL of 5% dextrose over two hours. Premedication: 100 mL of normal saline with corticosteroids, antiemetics, and H₂ blockers over 30 minutes.

Radiotherapy

Both groups received external beam radiotherapy, consisting of 66 Gray (Gy) delivered in 33 fractions over 6.5 weeks (2 Gy per fraction, five days per week). This treatment was administered using three-dimensional conformal radiation therapy on a 6 MV linear accelerator. Target delineation followed institutional guidelines, defining gross tumor volume, clinical target volume, and planning target volume with 5 mm margins.

Outcome measures

The primary outcome was treatment response at 6, 12, and 24 weeks post-treatment, assessed by the RECIST v1.1 [16,17] and categorized as complete response (CR), partial response (PR), stable disease (SD), or progressive disease (PD). Response evaluation included clinical examination, fiberoptic laryngoscopy, and imaging (CT/MRI) as indicated.

The secondary outcomes were acute and late treatment-related toxicities, graded according to CTCAE v3.0 [18], as this was the version in use at the time of study initiation and is widely reported in comparable HNC chemoradiotherapy studies. Acute toxicities were defined as occurring during treatment or within 90 days after completion, while late toxicities were those observed beyond 90 days.

Patient assessment at follow-up

Patients were monitored weekly during treatment to assess toxicity and compliance. Follow-up visits were scheduled at 6, 12, and 24 weeks post-treatment. At each visit, tumor response and toxicity were evaluated through physical examination, fiberoptic laryngoscopy, and imaging (CT or MRI) when indicated. Toxicities were recorded and categorized as acute (during treatment or ≤90 days after completion) or late (>90 days post-treatment).

Statistical analysis

Data were analyzed using SPSS version 25.0 (IBM Corp., Armonk, NY, USA). Categorical variables (treatment response, toxicity grades) were compared using chi-square or Fisher’s exact tests as appropriate (i.e., when expected cell counts were <5). For toxicity grading, we used the five-grade CTCAE v.3.0, which ranges from Grade 1 (mild) to Grade 5 (death), and was divided into two categories: clinically significant events (Grade 3-4) and lower-grade events (Grade 1-2) for comparative analysis. A p-value <0.05 was considered statistically significant. Analyses employed an intention-to-treat approach, which included all enrolled patients regardless of their adherence to the protocol or any treatment discontinuation.

## Results

**Figure 1:**
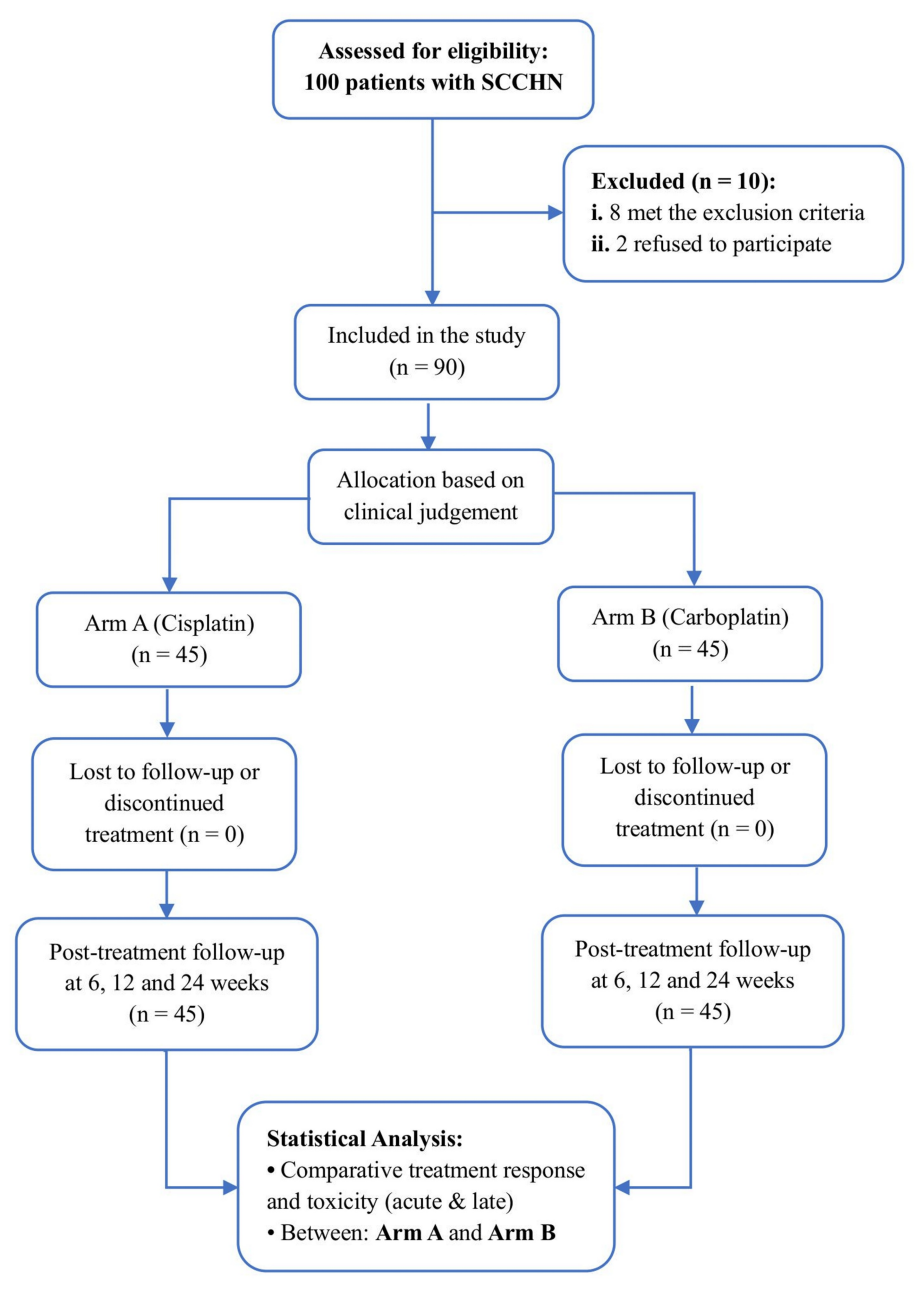
Study flowchart of patient enrollment, allocation, treatment, and follow-up. A total of 100 patients with SCCHN were screened, with 90 patients selected based on the required sample size. They were equally allocated to Arm A and Arm B; 10 were excluded (eight not eligible, two declined). All patients completed treatment and follow-up at 6, 12, and 24 weeks, with analyses comparing treatment response and toxicities between the arms. SCCHN = squamous cell carcinoma of the head and neck

Between June 2019 and December 2020, 100 patients with SCCHN were screened. Of these, 90 patients met the eligibility criteria and were equally allocated to Arm A (cisplatin, n = 45) and Arm B (carboplatin, n = 45). Allocation was determined by the treating oncologist based on clinical fitness parameters, including renal function, hematologic profile, and overall performance status (Figure 1).

Baseline characteristics

The mean age was 51.2 ± 5.3 years in the cisplatin group and 53.3 ± 4.6 years in the carboplatin group (p = 0.110). Most patients, 35 (77.8%) in each arm, were between 41 and 60 years old. The majority were male, with 37 (82.2%) in Arm A and 38 (84.4%) in Arm B.

Risk factor exposure was similar between groups. Smoking was reported by 33 (73.3%) versus 34 (75.6%), betel leaf chewing by 31 (68.9%) versus 29 (64.4%), and chewing tobacco by 27 (60.0%) versus 31 (68.9%) in Arm A and Arm B, respectively.

Most patients had an ECOG performance status of 0-1 (91.1% versus 88.9%). Baseline hematological parameters were also comparable between Arm A and Arm B, with mean hemoglobin levels of 12.2 ± 0.7 g/dL versus 12.2 ± 0.6 g/dL (p = 0.572), mean white blood cell counts of 5,752.7 ± 1,132.4 versus 5,684.7 ± 1,331.9 × 10³/mm³ (p = 0.202), and mean platelet counts of 178,214.1 ± 14,846.3 versus 175,564.6 ± 13,766.8 × 10³/mm³ (p = 0.402). No statistically significant differences were observed for any baseline demographic, clinical, risk factor, or hematological variable (all p > 0.05) (Table 1).

**Table 1:** Baseline demographic and clinical characteristics of study participants (n = 90). Values are presented as mean ± standard deviation (SD) or number (percentage). Risk factors included smoking (current or former for ≥6 consecutive months before diagnosis), betel leaf chewing (betel leaf with areca nut and lime, with or without tobacco), and chewing tobacco (smokeless oral tobacco). ¹: Independent t-test was applied for continuous variables; ²: Fisher’s exact test was used for categorical variables when expected cell counts were <5 (exact p-values reported, as no test statistic is generated); otherwise, ³: chi-square test was used. ECOG = Eastern Cooperative Oncology Group; WBC = white blood cell count; N/A = not applicable.

Variable	Arm A (n = 45)	Arm B (n = 45)	Test statistic	P-value
Demographics				
Age (years), mean ± SD	51.2 ± 5.34	53.25 ± 4.56	t = 1.63	0.110¹
Age group, n (%)				
40 or less	4 (8.9%)	2 (4.4%)	N/A	0.540²
41–50	15 (33.3%)	14 (31.1%)		
51–60	20 (44.4%)	21 (46.7%)		
Over 60	6 (13.3%)	8 (17.8%)		
Sex (male), n (%)	37 (82.2%)	38 (84.4%)	χ² = 0.13	0.720^3^
Risk factors				
Smoking, n (%)	33 (73.3%)	34 (75.6%)	χ² = 0.07	0.790^3^
Betel leaf chewing, n (%)	31 (68.9%)	29 (64.4%)	χ² = 0.18	0.671^3^
Chewing tobacco, n (%)	27 (60.0%)	31 (68.9%)	χ² = 0.79	0.373^3^
Clinical characteristics				
ECOG performance, n (%)				
0	18 (40.0%)	16 (35.6%)	N/A	0.310²
1	23 (51.1%)	24 (53.3%)		
2	4 (8.9%)	5 (11.1%)		
Hematology				
Hemoglobin (g/dL), mean ± SD	12.2 ± 0.7	12.2 ± 0.6	t = 0.57	0.572^1^
White blood cell count (×10³/mm³), mean ± SD	5,752.7 ± 1,132.4	5,684.7 ± 1,331.9	t = 1.29	0.202^1^
Platelet count (×10^3^/mm^3^), mean ± SD	178,214.1 ± 14,846.3	175,564.6 ± 13,766.8	t = 0.84	0.402^1^

Clinical profile of participants

The most common primary sites were the larynx (15 (33.3%) in Arm A and 16 (35.4%) in Arm B), hypopharynx (12 (26.7%) in Arm A and 14 (31.1%) in Arm B), and oropharynx (12 (26.7%) in Arm A and 10 (22.2%) in Arm B), with smaller proportions involving the oral cavity (6 (13.3%) in Arm A and 5 (11.1%) in Arm B). The majority of patients in both arms had advanced primary tumors (T3-T4a). In the cisplatin group, 18 (40.0%) had T3 disease and 18 (40.0%) had T4a disease, while in the carboplatin group, 17 (37.8%) had T3 and 20 (44.4%) had T4a tumors.

Nodal involvement was common, with most patients presenting with N1-N2 disease. In terms of overall stage grouping, stage III was observed in 19 (42.2%) patients in Arm A compared to 20 (44.4%) in Arm B. Stage IVA was found in 21 (46.7%) patients in Arm A versus 22 (48.9%) in Arm B.

Histologically, the majority of tumors were moderately differentiated, with 23 (51.2%) patients in Arm A and 20 (44.4%) in Arm B. This was followed by well-differentiated tumors, which accounted for 15 (33.3%) patients in Arm A and 17 (37.8%) in Arm B. Poorly differentiated tumors comprised 7 (15.6%) patients in Arm A and 8 (17.8%) in Arm B. There were no statistically significant differences between the two treatment arms for tumor site, T stage, nodal status, stage grouping, or histological grade (all p > 0.05) (Table 2).

**Table 2:** Tumor characteristics of study participants (n = 90). Values are presented as the number (percentage). No significant differences were observed between the cisplatin (Arm A) and carboplatin (Arm B) groups for tumor site, T stage, nodal stage, overall stage, or histological differentiation. ¹: Fisher’s exact test was used for categorical variables when expected cell counts were <5 (exact p-values reported, as no test statistic is generated); otherwise, ²: chi-square test was used. N/A = not applicable

Variable	Arm A (n = 45)	Arm B (n = 45)	Test statistic	P-value
Tumor site, n (%)				
Larynx	15 (33.3%)	16 (35.4%)	χ² = 1.78	0.067²
Oropharynx	12 (26.7%)	10 (22.2%)		
Hypopharynx	12 (26.7%)	14 (31.1%)		
Oral cavity	6 (13.3%)	5 (11.1%)		
T-stage (primary tumor), n (%)				
T2	4 (8.9%)	5 (11.1%)	N/A	0.412¹
T3	18 (40.0%)	17 (37.8%)		
T4a	18 (40.0%)	20 (44.4%)		
T4b	5 (11.1%)	3 (6.7%)		
Nodal stage (N-status), n (%)				
N0	4 (8.9%)	6 (13.3%)	N/A	0.712¹
N1	11 (24.4%)	11 (24.4%)		
N2A	8 (17.8%)	21 (46.7%)		
N2B	12 (26.7%)	7 (15.6%)		
N2C	10 (22.2%)	9 (20.0%)		
Stage group, n (%)				
III	19 (42.2%)	20 (44.4%)	N/A	0.230¹
IVA	21 (46.7%)	22 (48.9%)		
IVB	5 (11.1%)	3 (6.7%)		
Histological differentiation, n (%)				
Well-differentiated	15 (33.3%)	17 (37.8%)	χ² = 1.01	0.320²
Moderately differentiated	23 (51.2%)	20 (44.4%)		
Poorly differentiated	7 (15.6%)	8 (17.8%)		

Treatment response

At 6 weeks, CR was achieved in 17 (37.8%) patients in Arm A and 18 (40.0%) in Arm B, while PR was observed in 24 (53.3%) patients in each arm.

At 12 weeks, CR was documented in 18 (40.0%) patients in Arm A and 20 (44.4%) in Arm B; PR in 20 (44.4%) in Arm A and 21 (46.7%) in Arm B; SD in 4 (8.9%) in Arm A and 2 (4.4%) in Arm B; and PD in 3 (6.7%) in Arm A vs. 2 (4.4%) in Arm B.

At 24 weeks, CR was observed in 19 (42.2%) in Arm A and 22 (48.9%) in Arm B; PR in 21 (46.7%) in Arm A and 20 (44.4%) in Arm B; SD in 2 (4.4%) in Arm A and 1 (2.2%) in Arm B; and PD in 3 (6.7%) in Arm A and 2 (4.4%) in Arm B. There were no statistically significant differences in treatment response between the two arms at any follow-up interval (all p > 0.05) (Table 3).

**Table 3:** Treatment response at 6-, 12-, and 24-week follow-up intervals (n = 90). Values are presented as a number (percentage). Treatment response, assessed by the Response Evaluation Criteria in Solid Tumors version 1.1, did not differ significantly between the cisplatin (Arm A) and carboplatin (Arm B) groups at any time point. ¹: Fisher’s exact test was used for categorical variables when expected cell counts were <5 (exact p-values reported, as no test statistic is generated). CR = complete response; PR = partial response; SD = stable disease; PD = progressive disease; N/A = not applicable

Time point	Response type	Arm A (n = 45)	Arm B (n = 45)	Test statistic	P-value¹
6 weeks	CR, n (%)	17 (37.8%)	18 (40.0%)	N/A	0.094
	PR, n (%)	23 (51.1%)	24 (53.3%)		
	SD, n (%)	4 (8.9%)	3 (6.7%)		
12 weeks	CR, n (%)	18 (40.0%)	20 (44.4%)	N/A	0.149
	PR, n (%)	20 (44.4%)	21 (46.7%)		
	SD, n (%)	4 (8.9%)	2 (4.4%)		
	PD, n (%)	3 (6.7%)	2 (4.4%)		
24 weeks	CR, n (%)	19 (42.2%)	22 (48.9%)	N/A	0.486
	PR, n (%)	21 (46.7%)	20 (44.4%)		
	SD, n (%)	2 (4.4%)	1 (2.2%)		
	PD, n (%)	3 (6.7%)	2 (4.4%)		

Treatment response by histological differentiation

Among patients with well-differentiated tumors, CR was achieved in 10/15 (66.7%) in the cisplatin group and 11/17 (64.7%) in the carboplatin group. In moderately differentiated tumors, CR was seen in 6/23 (26.1%) and 7/20 (35.0%), respectively. Among poorly differentiated tumors, CR occurred in 3/7 (42.9%) and 4/8 (50.0%) patients, respectively. There were no statistically significant differences in CR rates between the two treatment groups across histological subtypes (all p > 0.05) (Table 4).

**Table 4:** Treatment response by histological differentiation. Values are presented as number/total (percentage). No significant differences were observed between the cisplatin (Arm A) and carboplatin (Arm B) groups across histological subtypes. ¹: Fisher’s exact test was used for categorical variables when expected cell counts were <5 (exact p-values reported, as no test statistic is generated); otherwise, ²: chi-square test was used CR = complete response; N/A = not applicable

Histological differentiation	CR - Arm A	CR - Arm B	Test statistic	P-value
Well-differentiated	10/15 (66.7%)	11/17 (64.7%)	χ² = 0.24	0.202²
Moderately differentiated	6/23 (26.1%)	7/20 (35.0%)	χ² = 0.53	0.313²
Poorly differentiated	3/7 (42.9%)	4/8 (50.0%)	N/A	0.215¹

Acute toxicity profile

Acute toxicity, with Grade ≥2 skin reactions, was reported in 23 (51.1%) patients in Arm A and 18 (40.0%) in Arm B. Oral mucositis of grade ≥2 occurred in 25 (55.6%) and 23 (51.1%) patients, and nausea/vomiting in 21 (46.7%) and 17 (37.8%) patients, respectively. Vomiting alone was reported in 17 (37.8%) and 12 (26.7%), respectively.

Hematological toxicities were less common. Neutropenia of grade ≥2 was documented in 5 (11.1%) and 9 (20.0%) patients, while thrombocytopenia of grade ≥2 occurred in 2 (4.4%) and 7 (15.6%) patients in Arm A and Arm B, respectively. The difference in thrombocytopenia was statistically significant (p = 0.040), whereas all other comparisons were not significant (p > 0.05) (Table 5).

**Table 5:** Acute toxicities during treatment and up to 90 days (Common Terminology Criteria for Adverse Events version 3.0). Values are presented as number (percentage). Grade ≥2 acute toxicities were generally comparable between groups, except for thrombocytopenia, which was significantly higher in the carboplatin arm. ¹: Fisher’s exact test was used for categorical variables when expected cell counts were <5 (exact p-values reported, as no test statistic is generated); otherwise, ²: chi-square test was used; *: p-values <0.05 considered statistically significant. N/A = not applicable

Toxicity type	Grade	Arm A (n = 45)	Arm B (n = 45)	Test statistic	P-value
Skin reaction, n (%)	Grade ≥2	23 (51.1%)	18 (40.0%)	χ² = 2.28	0.132²
Oral mucositis, n (%)	Grade ≥2	25 (55.6%)	23 (51.1%)	χ² = 0.62	0.432²
Nausea/Vomiting, n (%)	Grade ≥2	21 (46.7%)	17 (37.8%)	χ² = 1.34	0.247²
Vomiting, n (%)	Grade ≥2	17 (37.8%)	12 (26.7%)	χ² = 0.63	0.430²
Neutropenia, n (%)	Grade ≥2	5 (11.1%)	9 (20.0%)	χ² = 1.78	0.180²
Thrombocytopenia, n (%)	Grade ≥2	2 (4.4%)	7 (15.6%)	N/A	0.040*¹

Late toxicities

Grade ≥2 skin toxicity was reported in 6 (13.3%) patients in Arm A and 3 (6.7%) in Arm B. Grade 2 salivary gland toxicity was observed in 17 (37.8%) and 14 (31.1%) patients, respectively. Grade ≥2 dysphagia occurred in 10 (22.2%) and 8 (17.8%) patients, while Grade 2 mucositis was reported in 5 (11.1%) and 3 (6.7%) patients, respectively. There were no statistically significant differences in late toxicities between the two treatment groups (all p > 0.05) (Table 6).

**Table 6:** Late toxicities beyond 90 days (Common Terminology Criteria for Adverse Events version 3.0) Values are presented as a number (percentage). No statistically significant differences in late toxicities were observed between the cisplatin (Arm A) and carboplatin (Arm B) groups. ¹: Fisher’s exact test was used for categorical variables when expected cell counts were <5 (exact p-values reported, as no test statistic is generated); otherwise, ²: chi-square test was used. N/A = not applicable

Toxicity type	Grade	Arm A (n = 45)	Arm B (n = 45)	Test statistic	P-value
Skin toxicity, n (%)	Grade 2	6 (13.3%)	3 (6.7%)	N/A	0.563¹
Salivary gland, n (%)	Grade 2	17 (37.8%)	14 (31.1%)	χ² = 0.30	0.824²
Dysphagia, n (%)	Grade ≥2	10 (22.2%)	8 (17.8%)	χ² = 0.21	0.892²
Mucositis, n (%)	Grade 2	5 (11.1%)	3 (6.7%)	N/A	0.777¹

## Discussion

This quasi-experimental study compared weekly cisplatin (Arm A) and carboplatin (Arm B) as CCRT regimens in patients with LA-SCCHN. The baseline profiles of patients in both study arms were broadly comparable, with no statistically significant imbalances. The mean age was 51.2 ± 5.3 years in Arm A and 53.3 ± 4.6 years in Arm B (p = 0.110). Most participants were middle-aged, which aligns with the established epidemiology of head and neck squamous cell carcinoma (HNSCC). This cancer typically peaks during the fourth and sixth decades of life, with percentages ranging from 33.3% to 46.7% in these age groups. This age distribution is consistent with regional cancer registries and global GLOBOCAN data, which highlight the increasing incidence of HNSCC beyond the age of 40 years [1,19].

As is commonly observed in South Asian cohorts, there was a strong male predominance (73.3% vs. 75.6%), which mirrors known sex-specific differences in risk factor exposure, particularly smoking (73.3% vs. 75.6%), smokeless tobacco (60.0% vs. 68.9%), and betel nut (pan-supari) chewing (68.9% vs. 64.4%). The finding of strong male predominance in our cohort is consistent with national patterns, where men more frequently engage in tobacco chewing habits. The NICRH registry (2018-2020) reported that over half of male cancer patients were habituated to chewing tobacco, underscoring this association. However, data for other risk exposures (such as smoking or betel leaf chewing) are less detailed in the registry, making direct comparison more limited [20]. The similarity in exposure patterns between the two arms strengthens the internal validity of subsequent outcome comparisons.

Nearly 90% of patients in both arms had ECOG performance status 0-1 (91.1% vs. 88.9%) (p = 0.310), reflecting a treatment-fit cohort appropriate for CCRT, consistent with prior evidence highlighting the prognostic value of functional status in HNSCC [3,21]. Socioeconomic status was mainly middle-tier in both arms (60.0% vs. 53.3% of patients), which is significant in the South Asian context, where lower socioeconomic status often delays diagnosis and hampers adherence to complex treatment plans [12,19]. The similarity of baseline variables between groups suggests that any later differences in efficacy or toxicity are more likely due to treatment rather than underlying demographic or clinical differences.

The most frequent tumor sites were the larynx (Arm A: 33.3%, Arm B: 35.4%), hypopharynx (~29%), and oropharynx (~24%), with fewer oral cavity tumors (~12%). This distribution mirrors South Asian epidemiology, where upper aerodigestive tract cancers predominate due to tobacco and betel quid use. However, oral cavity cancers are often more common in other national registries [2,3].

Most patients presented with advanced primary tumors (T3-T4a in nearly 80% for both arms) and nodal involvement, resulting in stage III-IVa disease in almost 90% of cases. Such late presentation is consistent with regional data and reflects diagnostic delays driven by socioeconomic and health system barriers [21]. Comparable findings are documented in Bangladesh’s NICRH registry and in neighboring South Asian centers [20].

Histologically, the majority of tumors were moderately differentiated (Arm A: 51.2%; Arm B: 44.4%), followed by well (Arm A: 33.3% Arm B: 37.8%) and poorly differentiated (Arm A: 15.6% Arm B: 17.8%) carcinomas, aligning with global patterns for HNSCC [20,22]. Importantly, no significant imbalances were observed between arms in terms of site, stage, or histological grade, ensuring comparability for treatment outcomes.

At six weeks, CR and PR rates were high in both arms, with CR observed in nearly 40% of patients and PR in just over half. This early tumor regression demonstrates effective radiosensitization by both agents, consistent with prior studies of weekly cisplatin and carboplatin showing response rates exceeding 80% at first assessment [23,24].

By 12 weeks, CR rates increased slightly to 40.0% in Arm A and 44.4% Arm B, with most remaining patients achieving PR (Arm A: 44.4%, Arm B: 46.8%). Only a small minority demonstrated SD or PD, accounting for approximately 4-7% in both arms, highlighting that both regimens achieved robust early locoregional control. Comparable findings have been reported in matched-pair and retrospective analyses, where carboplatin was not inferior to cisplatin in short-term response [24,25].

At 24 weeks, CR rates stabilized at 48.9% in Arm A and 42.2% in Arm B, with combined CR + PR remaining above 85% in both groups. A small proportion of patients exhibited SD or PD, with rates ranging from 4% to 7%. The lack of significant differences at this interval reinforces prior evidence that weekly carboplatin can provide similar short-term efficacy to cisplatin, particularly in real-world or comorbidity-laden populations [26,27]. However, these response data reflect only early disease control, and longer follow-up is needed to determine whether equivalence extends to progression-free or overall survival.

Similarly, in terms of treatment responses stratified by histological differentiation, both regimens (Arm A and Arm B) produced comparable CR rates across well-, moderately, and poorly differentiated tumors, with no statistically significant differences between cisplatin (Arm A) and carboplatin (Arm B) (all p > 0.05). This finding also suggests that the short-term radiosensitizing effect of weekly platinum therapy was broadly consistent regardless of differentiation, aligning with prior comparative studies showing similar locoregional response to cisplatin and carboplatin across heterogeneous tumor biology [3,22,25].

Toxicity patterns were generally comparable. Acute mucocutaneous toxicities were more prominent with cisplatin (Arm A: 51.1% vs. Arm B: 40.0%). In contrast, gastrointestinal toxicities, including mucositis, skin reactions, and nausea/vomiting, were common in both regimens and did not differ significantly. This aligns with published CRT series that report high rates of Grade ≥2 mucositis and dermatitis during combined-modality treatment [25]. In contrast, carboplatin resulted in greater hematologic suppression, particularly a higher rate of Grade ≥2 thrombocytopenia (Arm A: 4.4% vs. Arm B: 15.6%), which was statistically significant and has been documented in clinical studies [11]. Although nephro- and ototoxicity were not the dominant acute events in our trial, these remain classic dose-limiting toxicities of cisplatin, as shown in recent comparative studies examining nephrotoxicity [28] and ototoxicity [6,29].

Late toxicities, including dysphagia and salivary gland dysfunction, were comparable between cisplatin and carboplatin, consistent with prior reports showing that radiation-related late effects remain similar irrespective of concurrent platinum agent [25,27]. These sequelae, particularly swallowing dysfunction, are well described in the literature and may persist or emerge months after treatment [30]. Comparative analyses of platinum-based concomitant regimens have generally found that the addition of chemotherapy increases acute mucosal toxicity but has a less consistent impact on late functional outcomes. Head-to-head studies of cisplatin versus carboplatin have not shown major differences in late toxicity profiles at early follow-up [2,3,25].

Limitations

This study has several limitations. Its single-center, quasi-experimental, non-randomized design with a relatively small sample size may limit generalizability and introduce selection bias, as patients were not allocated randomly or matched by baseline characteristics. Additionally, the lack of long-term survival and quality-of-life outcomes restricts conclusions about durability and patient-centered impacts. Nonetheless, the prospective design and consistent treatment protocols strengthen the methodological rigor. Future randomized controlled trials with larger, multi-center cohorts and longer follow-up are necessary to validate and expand these findings.

## Conclusions

This quasi-experimental study suggests that weekly carboplatin is a clinically feasible and well-tolerated alternative to weekly cisplatin in CCRT for LA-SCCHN. Both regimens achieved comparable tumor response and late toxicity outcomes. Cisplatin was associated with higher rates of mucocutaneous and gastrointestinal toxicities, whereas carboplatin carried a greater risk of hematologic adverse events. These findings provide preliminary evidence supporting carboplatin as a reasonable option for patients unsuitable for cisplatin, provided that careful toxicity monitoring and supportive care are implemented. Larger randomized trials with long-term follow-up are warranted to confirm these results and strengthen clinical recommendations.
